# Cancer-Associated Carbohydrate Antigens as Potential Biomarkers for Hepatocellular Carcinoma

**DOI:** 10.1371/journal.pone.0039466

**Published:** 2012-07-13

**Authors:** Chen-Shiou Wu, Chia-Jui Yen, Ruey-Hwang Chou, Shiou-Ting Li, Wei-Chien Huang, Chien-Tai Ren, Chung-Yi Wu, Yung-Luen Yu

**Affiliations:** 1 The Ph.D. Program for Cancer Biology and Drug Discovery, China Medical University, Taichung, Taiwan; 2 Graduate Institute of Cancer Biology and Center for Molecular Medicine, China Medical University, Taichung, Taiwan; 3 Graduate Institute of Clinical Medicine, National Cheng Kung University, Tainan, Taiwan; 4 Division of Hematology/Oncology, Department of Internal Medicine, National Cheng Kung University Hospital, Tainan, Taiwan; 5 Genomics Research Center, Academia Sinica, Taipei, Taiwan; 6 Department of Biotechnology, Asia University, Taichung, Taiwan; University of Modena & Reggio Emilia, Italy

## Abstract

Hepatocellular carcinoma (HCC) is one of the most common human malignancies. Therefore, developing the early, high-sensitivity diagnostic biomarkers to prevent HCC is urgently needed. Serum a-fetoprotein (AFP), the clinical biomarker in current use, is elevated in only ∼60% of patients with HCC; therefore, identification of additional biomarkers is expected to have a significant impact on public health. In this study, we used glycan microarray analysis to explore the potential diagnostic value of several cancer-associated carbohydrate antigens (CACAs) as biomarkers for HCC. We used glycan microarray analysis with 58 different glycan analogs for quantitative comparison of 593 human serum samples (293 HCC samples; 133 chronic hepatitis B virus (HBV) infection samples, 134 chronic hepatitis C virus (HCV) infection samples, and 33 healthy donor samples) to explore the diagnostic possibility of serum antibody changes as biomarkers for HCC. Serum concentrations of anti-disialosyl galactosyl globoside (DSGG), anti-fucosyl GM1 and anti-Gb2 were significantly higher in patients with HCC than in chronic HBV infection individuals not in chronic HCV infection patients. Overall, in our study population, the biomarker candidates DSGG, fucosyl GM1 and Gb2 of CACAs achieved better predictive sensitivity than AFP. We identified potential biomarkers suitable for early detection of HCC. Glycan microarray analysis provides a powerful tool for high-sensitivity and high-throughput detection of serum antibodies against CACAs, which may be valuable serum biomarkers for the early detection of persons at high risk for HCC.

## Introduction

Hepatocellular carcinoma (HCC) is the fifth most common cancer worldwide, with China and North America showing a continuous increase in the incidence and mortality rate [1]. HCC nearly always develops in the setting of chronic hepatitis virus infection or liver cirrhosis [2]–[4]. The prognosis for patients with HCC remains poor, and the 5-year survival rate after diagnosis OR for most patients is less than 5%, mainly because the disease is often diagnosed in an advanced stage [5]. For patients with a diagnosis of HCC at an early stage, the survival rate can be improved significantly by surgical resection, liver transplantation, and other curative therapies such as ablative treatments [6], [7]. Moreover, surveillance of at-risk patients improves detection and potentially the curative effect of treatments for small tumors. Therefore, early prognostic markers are crucial for effective treatment and prevention of HCC.

The most common HCC biomarker used to screen patients with liver cirrhosis is serum a-fetoprotein (AFP), which is measured at 6-month intervals [8]. Nevertheless, AFP levels are often elevated in some patients with chronic liver disease who do not have cancer, and AFP levels are not elevated in 30–40% of patients with liver cancer [9]. The serum AFP test has low sensitivity, and about one-third of patients with early-stage HCC and small tumors (<3 cm) have the same level of AFP as that in normal individuals, which makes the AFP test insufficient for the early detection of HCC in at-risk populations [10]. In addition, the AFP test has a high false-positive rate of ∼20% among patients with chronic hepatitis and 20–50% among those with liver cirrhosis [5], [11]. In this regard, there is an urgent need to identify more sensitive and reliable serum biomarkers for the detection of HCC [12], [13].

Oncogenesis is often associated with changes in the expression of cell surface carbohydrates. In some instances, the carbohydrate pattern may be specific to the disease type [14]. In other instances, levels of anti-carbohydrate antibodies may be markedly enhanced with the onset of disease [15]. Previous studies have shown that cellular glycosylation profiles change significantly during carcinogenesis [14]. Carbohydrates play crucial roles in various biological events such as cell recognition [16], inter- and intracellular signaling, embryonic development, cell adhesion [17], and cell-cell interactions [18]. Currently, glycan marker discovery with glycan microarray analysis presents great potential for identifying biomarkers relevant for the diagnosis of breast cancer [19].

Glycan microarrays allow direct characterization of carbohydrate-protein interactions [20]. Microarray techniques are effective and sensitive methods for the rapid analysis of the specificity of protein-carbohydrate interactions and the characterization of differentiation processes pertaining to the onset of cancer at the molecular level [21]. In addition, the attachment of sugars to surfaces can effectively mimic the presentation of these compounds on the membrane of cells and thus can be used to bind antibodies [20]. In this report, we focused on glycans that are known to be cancer-associated carbohydrate antigens (CACAs) in many cancers but that have not been studied in HCC. We used glycan microarray analysis to explore the diagnostic possibility of serum antibody changes as biomarkers for HCC. In addition, we compared the accuracy of the biomarkers we identified with the conventional AFP biomarker for HCC.

## Results

### Patient Characteristics

A total of 593 participants including 293 HCC patients, 133 chronic hepatitis B virus (HBV) infection patients, 134 chronic hepatitis C virus (HCV) infection patients, and 33 normal subjects were recruited into this study (**Table 1**). There were no significant differences of age and sex between cases and controls. In addition, the HCC group and the healthy controls group had statistically different laboratory results for albumin, aspartate aminotransferase (AST), alanine aminotransferase (ALT) and total bilirubin (BIL-T) (p<0.05). The pathological staging (AJCC) of HCC was grade I in 96 cases (41.3%), grade II in 80 cases (34.5%), grade III in 48 cases (20.7%), and grade IV in 8 cases (3.4%).

**Table 1:** Summary of clinical details of subjects used for glycan microarray analysis.

	Healthy(n = 33)	CLD (HBV)(n = 133)	CLD (HCV)(n = 134)	HCC(N–293)
Men, n (%)	22 (66.7)	89 (66.9)	73 (54.5)	223 (76.1)
Laboratory values mean(SD)				
ALBUMIN(g/dL)	4.3 (0.2)	3.9 (0.6)	3.9 (0.5)	4.1 (0.6)
AST(U/L)	27.6 (10.3)	106.1 (144.8)	162.2 (514.6)	136.3 (241.3)
ALT(U/L)	29.3 (19.6)	152.2 (223.6)	185.2 (391.9)	119.4 (232.6)
BIL-T(mg/dL)	0.5 (0.1)	1.2 (1.7)	1.4(3.1)	0.7 (0.6)
Pathological staging(AJCC) (n = 232), n (%)				
I				96 (41.3)
II				80 (34.5)
III				48 (20.7)
IV				8 (3.4)

(CLD: chronic liver disease).

### Detection of Serum Antibodies Against Carbohydrate Antigens with Glycan Microarray Analysis

**Figure 1:**
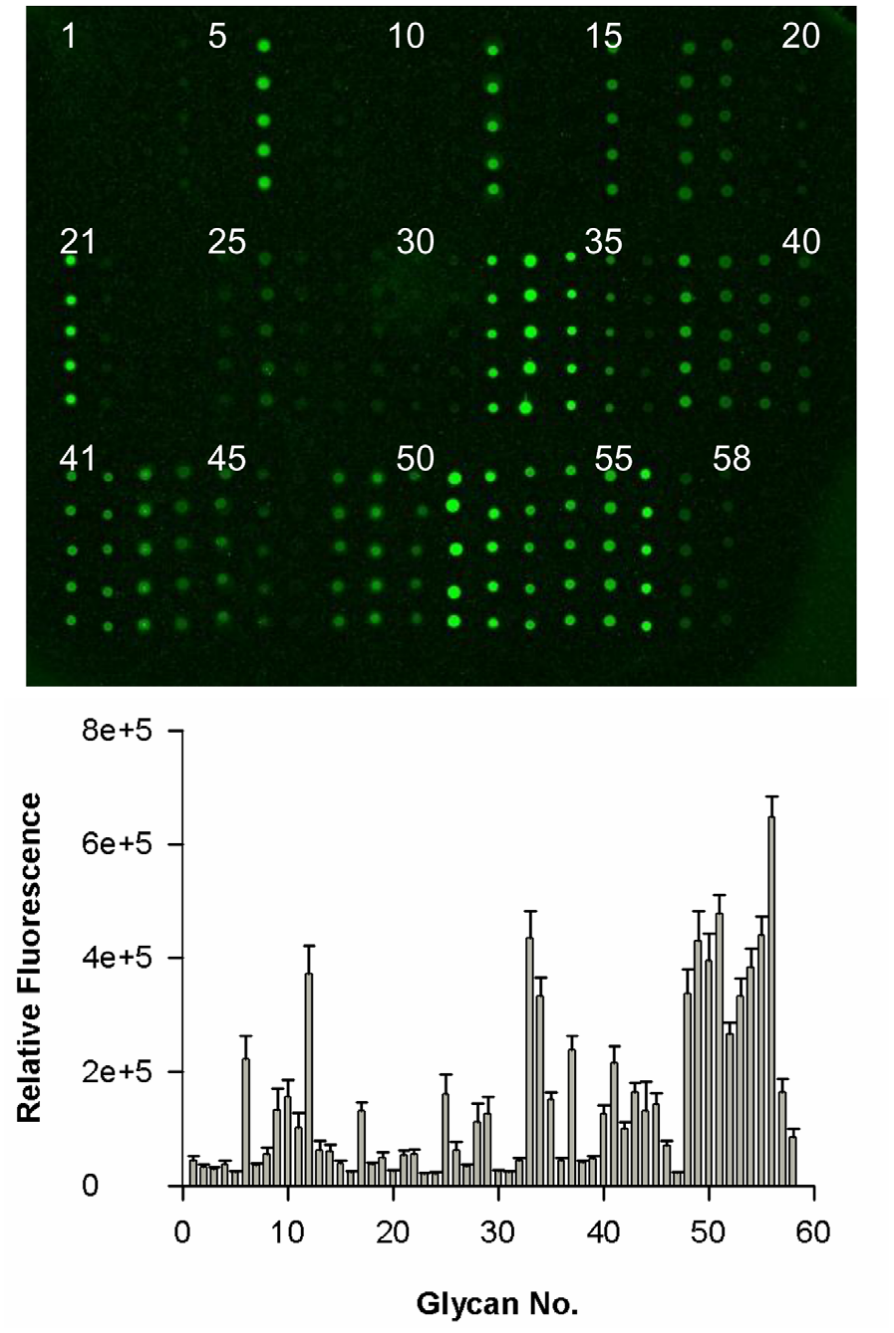
Detection of antibodies to 58 carbohydrate antigens in serum from patients with HCC. (A) A representative slide image obtained from a fluorescence scan after detection of IgG in serum samples. Each grid was printed with glycan numbers 1–20 in the top row, 21–40 in the middle row, and 41–58 in the bottom row. (B) Mean ± standard deviation binding specificity of antibodies to glycan numbers 1–58 in serum samples from patients with HCC. Relative fluorescence intensities were calculated for each spot (containing 100 µM glycan) in the glycan microarray.

We tested serum from patients with HCC for antibodies that bind to glycan fragments. Glass slides printed in microarray format with glycan compounds 1–58 (**Figure S1**) were incubated with serum samples, and bound antibodies were detected using Cy3-labeled goat anti–human IgG secondary antibody (**Figure 1**). The fluorescent scans were processed to quantify the intensity of each spot. The resulting data were represented as the mean relative fluorescence intensity from replicates of each sample. We noted that the background intensity of carbohydrate antigen binding from healthy individuals could be the result of non-specific binding; therefore, mean fluorescence intensities of <35,000 were removed from the analysis. This approach provided a rationale for the development of a glycan microarray to detect the presence of 47 different glycan antibodies in the serum of patients (after removal of sample numbers 2, 3, 5, 7, 16, 20, 23, 24, 30, 31, and 47).

**Figure 2:**
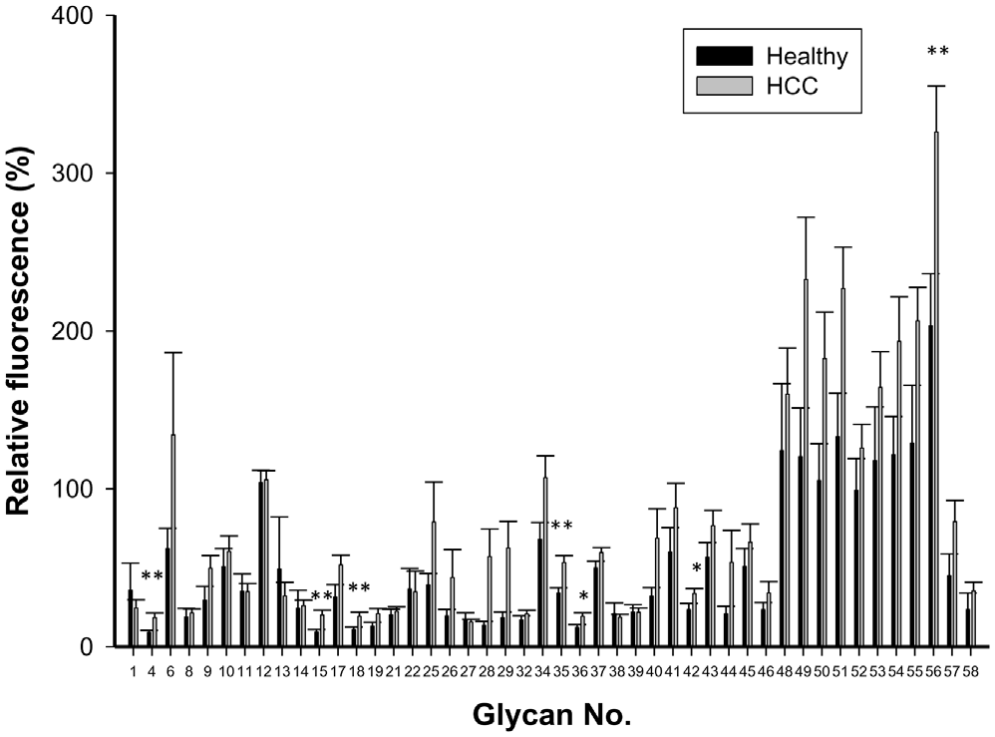
Ratio of anti-glycan IgG levels to anti-Gb5 levels in serum from healthy individuals and patients with HCC. Relative fluorescence ratios were calculated as the relative fluorescence intensity of each glycan analog divided by the relative fluorescence intensity of Gb5, and they are expressed as percentages in the figure. The figure shows the mean ratio ± standard deviation for each glycan. Asterisks indicate glycans in which the mean glycan:Gb5 IgG ratio was significantly higher in serum from patients with HCC (n = 155) than in serum from healthy individuals (n = 33). The *p* values were calculated with a Student’s *t*-test (*p<0.05; **p<0.01).

We examined serum samples from 155 patients with HCC and 33 healthy individuals for antibodies that bound to carbohydrate antigens on the glycan microarray. The fluorescence data reflecting antibody reactivity to each glycan were normalized to Gb5 for each sample, and the relative fluorescence intensities for IgG antibodies from patients with HCC and healthy individuals are presented as mean glycan:Gb5 IgG ratios (**Figure 2**). Antibodies that bound to Gb5 were detected in patients with HCC and healthy donors. We observed that the levels of IgG against glycan numbers 4 (SLacNAc 6SO_3_), 15 (disialosyl galactosyl-globoside; DSGG), 18 (fucosyl GM1), 35 (Gb3), 36 (Gb2), 42 (B19), and 56 (Man7) were significantly higher in patients with HCC than in healthy individuals.

### Comparison of the Levels of Seven Anti-glycan Antibodies Among Female and Male Patients with HCC and Healthy Individuals

**Figure 3:**
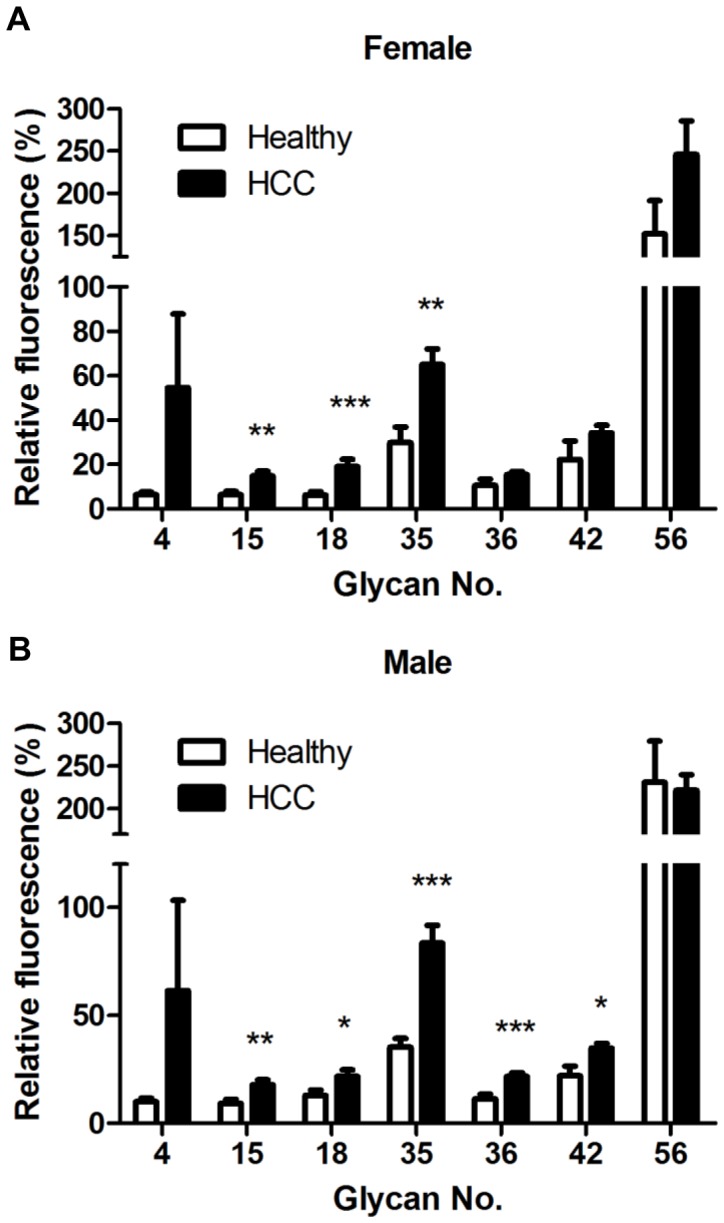
Comparison of the level of seven anti-glycan antibodies in serum from healthy individuals and patients with HCC for females and males. Relative fluorescence ratios were calculated as the fluorescence intensity for each glycan analog divided by the fluorescence intensity for Gb5, and they are expressed as percentages in the figures. The figure shows the mean ratio ± standard deviation for each glycan. (A) The glycan:Gb5 IgG ratios for glycan numbers 15, 18, and 35 were significantly higher in serum from female patients with HCC (n = 70) than in serum from healthy females (n = 11). (B) The glycan:Gb5 IgG ratios for glycan numbers 15, 18, 35, 36 and 42 were significantly higher in serum from male patients with HCC (n = 223) than in serum from healthy males (n = 22). Asterisks indicate glycans in which the mean glycan:Gb5 IgG ratio was significantly higher in serum from patients with HCC than in serum from healthy individuals. The *p* values were calculated with a Student’s *t*-test (*P<0.01).

In a previous study [22], men had a higher incidence of HCC than women. In our current study, we increased the HCC samples numbers up to 293 patients and stratified our results by gender to determine whether anti-glycan antibody levels varied by gender. Among females, levels of IgG against glycan numbers 15, 18, and 35 were significantly higher in patients with HCC than in healthy individuals (*P<*0.01) (**Figure 3A**). Among males, levels of IgG against glycan numbers 15, 18, 35, 36, and 42 were significantly higher in patients with HCC than in healthy individuals (*P<*0.05) (**Figure 3B**). Interestingly, we observed similar results for glycan numbers 15, 18 and 35 between two groups.

### Comparison of the Level of Select Anti-glycan Antibodies from HCC and Chronic Liver Disease (CLD) by Gender

**Figure 4:**
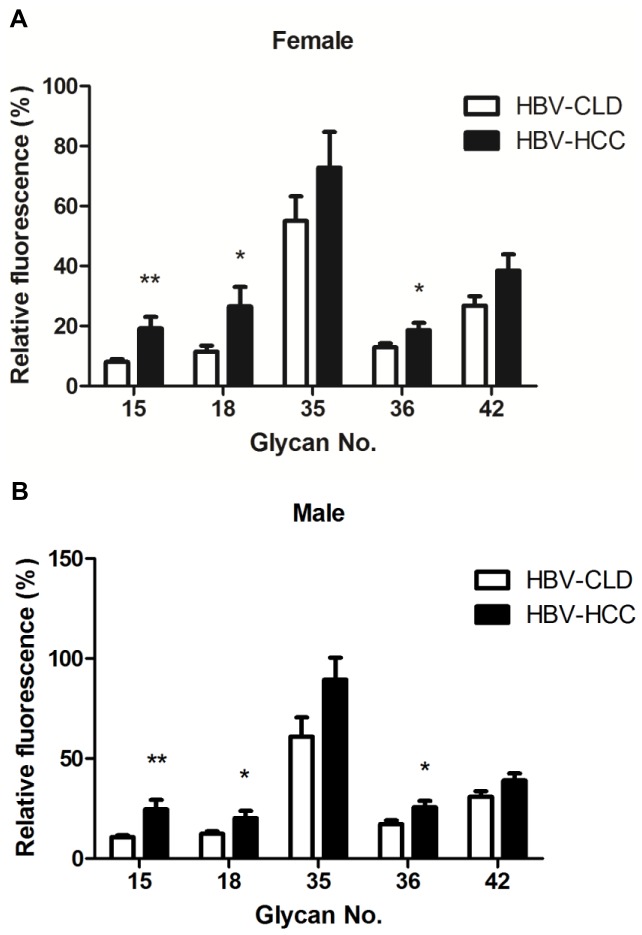
Comparison of the level of select anti-glycan antibodies from HBV-related HCC and CLD by gender. Relative fluorescence ratios were calculated as the fluorescence intensity for each glycan analog divided by the fluorescence intensity for Gb5, and they are expressed as percentages in the figures. The figure shows the mean ratio ± standard deviation for each glycan. The ratios for glycan numbers 15, 18, and 36 were significantly higher from HBV-related HCC than CLD (HBV) in females (n = 30 and 44, separately) (A) and males (n = 102 and 89, separately) (B). The *p* values were calculated with a Student’s *t*-test (*P<0.05, **P<0.01, ***P<0.001).

**Figure 5:**
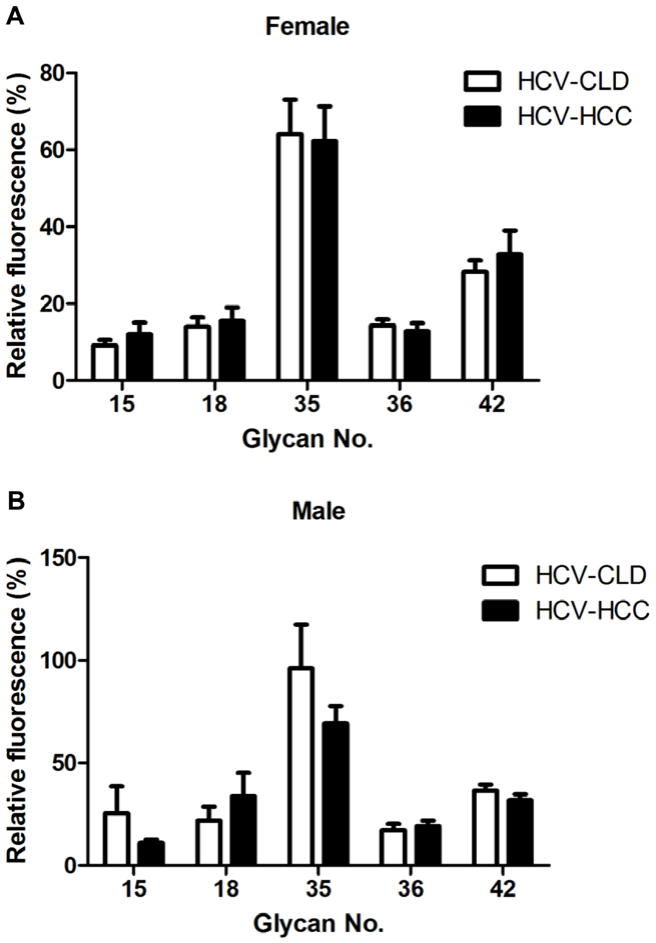
Comparison of the level of select anti-glycan antibodies from HCV-related HCC and CLD by gender. Relative fluorescence ratios were calculated as the fluorescence intensity for each glycan analog divided by the fluorescence intensity for Gb5, and they are expressed as percentages in the figures. The figure shows the mean ratio ± standard deviation for each glycan. The ratios for glycan numbers 15, 18, 35, 36 and 42 were no significant difference between HCV-related HCC and CLD (HCV) in females (n = 18 and 61, separately) (A) and males (n = 47 and 73, separately) (B). (*P*>0.05).

Worldwide, more than 52% of HCC cases are associated with chronic HBV infection, and 25% of HCC cases are associated with HCV infection [23]. Known HBV or HCV infection may increase the risk of developing HCC [7]. To examine differences in HCC patients with HBV or HCV infection, we divided patients into the HBV-positive HCC (HBV-HCC; n = 132) or HCV-positive HCC (HCV-HCC; n = 65) to compare chronic liver disease (HBV-CLD; n = 133 and HCV-CLD; n = 134). For both genders, levels of IgG against glycans 15, 18, and 36 for females and males were higher in HBV patients with HCC from those patients without HCC (*P*<0.05) (**Figure 4A and B**). There were no significant differences in anti-glycan levels between patients with HCV-HCC and chronic HCV patients (**Figure 5A and B**).

We next compared detection of glycan analogs and AFP for their diagnostic sensitivity for HCC from CLD and Healthy. For a patient with a history of liver disease, consensus for the diagnosis of HCC can be achieved with an elevated AFP level (≥200 ng/dL), along with a dynamic imaging study showing a hepatic tumor. In Taiwan, the cutoff value of serum AFP level for HCC diagnosis is set at 200 ng/ml, and according to the guidelines of clinical diagnosis and staging criteria for HCC. Therefore, we change cutoff value of AFP. When AFP_200_ (AFP at a cutoff value of >200 ng/ml) was used to predict the HCC stage, a sensitivity of 33.33% (10 of 30) was obtained for females, and 24.51% (25 of 102) was obtained for males (**Table 2**). We observed that the sensitivity of 3 anti-glycan numbers (15, 18, and 36) were better than that of AFP to 63.33% (19 of 30)/42.16% (43 of 102), 56.67% (17 of 30)/35.29% (36 of 102), and 43.33% (13 of 30)/45.1% (46 of 102), respectively, in female/male. (**Table 2**). To test whether the sensitivity of classification could be increased by including clinical data, AFP values were included in the analysis. As seen in Table 2, inclusion of AFP values and 3 anti-glycan markers increased sensitivity of the correct classification of HCC states to 80% (24 of 30) and 74.5% (76 of 102), respectively, in female and male.

**Table 2:** Comparison of the sensitivity of select anti-glycan antibodies and AFP for the diagnosis of HBV-positive HCC in females and males.

Glycan female	Sensitivity (%)	Glycan male	Sensitivity (%)
15(DSGG)	63.33%	15(DSGG)	42.16%
18(Fucosyl GM1)	56.67%	18(Fucosyl GM1)	35.29%
36(Gb2)	43.33%	36(Gb2)	45.10%
AFP_200_	33.33%	AFP_200_	24.51%
(15)+(18)+(36)+AFP_200_	80%	(15)+(18)+(36)+AFP_200_	74.51%

NOTE: AFP_200,_ AFP at a cutoff value >200 ng/ml.

### The Diagnostic Value of Three Anti-glycan Antibodies for HCC

**Figure 6:**
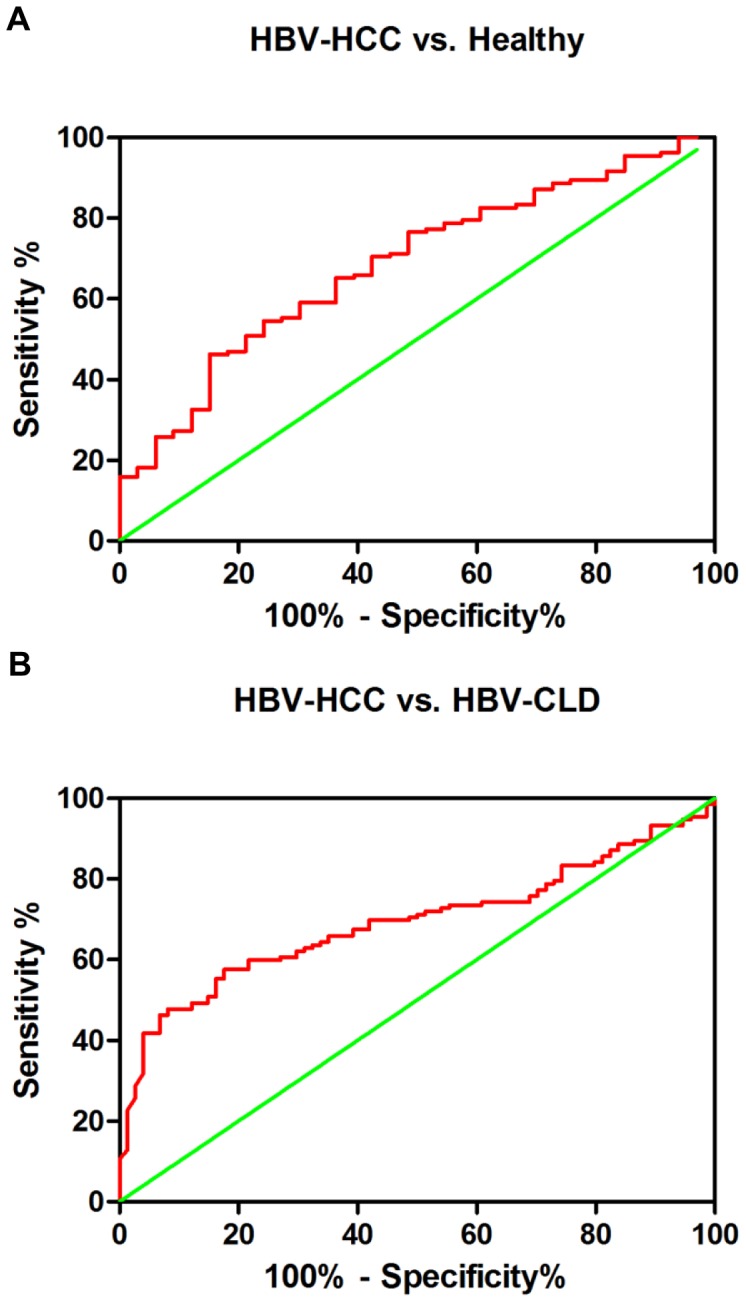
ROC curve analysis using serum anti-glycan antibodies for discriminating HCC patients from HBV-CLD patients and healthy controls. These 3 anti-glycan antibodies yielded an AUC (the areas under the ROC curve) of 0.6843 (95% CI: 0.5882–0.7805) with 46.21% sensitivity and 84.85% specificity for discriminating HCC patients from healthy controls (A), and an AUC of 0.6923 (95% CI: 0.6215–0.7631) with 57.58% sensitivity and 82.43% specificity for discriminating HCC from HBV-CLD patients (B).

To evaluate whether serum anti-glycan levels can be used as a potential diagnostic marker for HCC, Receiver operating characteristics (ROC) curve analyses were performed. It was revealed that serum 3 anti-glycan markers plus AFP levels was a potential marker for discriminating HBV-positive HCC patients from healthy controls with an AUC (the areas under the ROC curve) of 0.6843 (95% CI: 0.5882–0.7805) (**Figure 6A**). At the cut-off value of 0.4974, the sensitivity and specificity for this marker was 46.21% and 84.85%. The AUC of serum 3 anti-glycan markers plus AFP levels for discriminating HBV-positive HCC from chronic HBV was 0.6923 (95% CI: 0.6215–0.7631) (**Figure 6B**). At the cut-off value of 0.5268, the sensitivity and specificity for this marker was 57.58% and 82.43%. This result suggests that these 3 anti-glycan markers plus AFP may be applicable to the diagnosis of HBV-related HCC.

## Discussion

Tumor markers in the serum of patients with HCC can be used as diagnostic tools, prognostic factors, and treatment parameters [24]. Using our glycan microarray, we examined serum to search for antibodies that could distinguish between patients with HCC and healthy individuals. This study has demonstrated the potential of a glycan microarray for identifying CACAs biomarker candidates in serum from patients with HCC. In our current study, the differences in serum antibody levels between patients with HCC and healthy individuals were substantial, with antibody levels for 7 of 58 glycans differentially abundant (p<0.01). These glycans were SLacNAc 6SO_3_, DSGG, fucosyl GM1, Gb3, Gb2, B19, and Man7. Alterations in glycosylation in cancer, as well as other diseases, have attracted much interest and have shown potential both as a source of disease markers and as targets for immunotherapy of tumors [15]. Changes in glycosyltransferase expression levels commonly lead to an increase in the size and branching of N-linked glycans, which creates additional sites for terminal sialic acid residues [25]. A corresponding increase in sialyltransferase expression ultimately leads to an overall increase in sialylation [26]. Overexpression of the glycosyltransferases that are involved in linking terminal residues to glycans leads to overexpression of certain terminal glycan epitopes such as SLacNAc 6SO_3_, DSGG, and fucosyl GM1 on tumors [27]–[29].

We found that anti-DSGG levels distinguished between patients with HCC and CLD. DSGG is a disialyl glycosphingolipid with a globo-series core structure [27]. In a previous study, DSGG was identified as an adhesion molecule expressed on renal cell carcinoma, and its relationship to metastatic cancer was demonstrated [27]. Our data are the first to suggest that DSGG is highly expressed in HCC.

Interestingly, a gender difference has been described for HCC in almost all study populations, with a male:female ratio for the incidence of HCC averaging between 2∶1 and 4∶1 [30]. One mechanism that may explain the gender disparity observed in the incidence of HCC is the increased activity of estrogens in female patients, which may offer protection from hepatocarcinogenesis [22]. In addition, there are probably other cellular regulatory molecules involved in the gender disparity in the incidence of HCC. In our current study, we observed a gender disparity in levels of slightly different anti-carbohydrate antibodies. Levels of anti-fucosyl GM1 differed in patients with HCC and chronic HBV or healthy individuals. Fucosyl GM1 belongs to one kind of gangliosides [28]. In a previous study, fucosyl GM1 was strongly associated with small-cell cancer of the lung, and this tumor-associated antigen was detected with high sensitivity and specificity using an immunofluorescence method [28]. The role of this glycan in cancer is unclear. The glycosylation of hormone receptors has been suggested as an explanation for gender disparities in coronary heart disease and prostate cancer [31], [32]. Although how general these observations are to populations with a more heterogeneous disease etiology remains to be examined, the results presented here on potential glycan biomarkers seem quite encouraging. Recently, large-scale analytical methods have been developed for the human serum glycoproteome, which is also a powerful tool for the discovery of diagnostic and therapeutic targets. Using lectin-based glycoproteomic analysis, glycoprotein (GP) 73 was found to be a novel tumor marker for HCC [33]. Serum GP73 levels were significantly increased in patients with HCC, even in HCC patients with serum AFP levels less than 20 ng/ml [24]. Fucosylation of GP73 is reported to be increased in patients with HCC [33]. A previous study suggested that fucosylation plays an important role in the interaction between interleukin-8 and its receptor, thereby inducing the migration of cancer cells in HCC [34]. Our current results suggest that fucosyl GM1 is a candidate hepatic CACAs biomarkers.

HCC is associated with HBV infection in approximately 50% of cases [35]. In this study, we observed that five types of anti-glycan antibodies were increased in patients with HCC and HBV infection. Therefore, we extrapolate that the increase in antibodies and HBV infection are related. Woodchucks are used as a preferred animal model of chronic HBV infection [36]. This model recapitulates the disease progression of HBV infection to HCC and has documented similarities in protein glycosylation with human HCC [36]. As HCC usually takes years to develop as a result of chronic HBV infection, investigation of early events in the woodchuck model of HBV infection may provide helpful information about potential biomarkers for the prognosis of HCC. The biological influences driving these changes require further examination.

AFP is the only serological marker currently used for clinical detection of HCC. Although AFP improves the detection of HCC, a significant number of patients with HCC do not have elevated AFP levels, and thus, additional biomarkers are needed to increase the sensitivity of HCC detection [37], [38]. In past research, AFP has shown limited sensitivity (41–65%) in the diagnosis of HCC [39]. Therefore, there is an urgent need to discover additional biomarkers for the screening and diagnosis of HCC. Our study compared patients with HCC and CLD plus healthy, and these 3 glycan provided better sensitivity than AFP. Moreover, the sensitivity of 3 anti-carbohydrate antibodies plus AFP provided better diagnosis sensitivity.

In addition, there was no correlation between levels of these 3 anti-glycan antibodies and the tumor stage (data not shown). Therefore, we suggest that the 3 anti-carbohydrate antibodies may be as biomarkers for the initiation of tumor formation, but not tumor progression. Further examination using a larger patient group and a validation process that includes prospectively collected patient samples are needed to verify our observations. These new CACAs biomarkers, in combination with other existing serologic biomarkers, can be valuable in the diagnosis of HCC. Based on our results, we suggest that combining glycan microarray analysis with AFP measurements will provide a much better tool for HCC diagnosis than the use of AFP alone.

Biomarkers have long been sought for their diagnostic, prognostic, and therapeutic uses. However, there are no reliable biomarkers for most solid tumors, including HCC. The methods described in our current study are useful for identifying possible biomarkers of disease. The development of a glycan microarray has enabled high-sensitivity and high-throughput analysis of carbohydrate-protein interactions and has contributed to significant advances in glycomics. This approach provides both a powerful tool for basic research and a promising technique for medical diagnosis and the detection of pathogens and cancers [20]. This method requires very small amounts of material and is more effective and sensitive than the traditional ELISA method; thus, it provides another platform to monitor the immune response to carbohydrate epitopes at different stages during differentiation, metastasis, and treatment [21]. Our current research identified five CACAs biomarkers for HCC diagnosis that need to be evaluated further in future work. Identification of CACAs will be useful in the development of new cancer therapies such as generation of vaccines or antibodies targeting CACAs [14], [19], [40]–[42]. We believe that the glycan microarray is a viable method for clinical diagnosis of HCC. This method should improve prognosis and may offer insights into the development of novel therapeutic approaches for other cancers. New glycan antigens may prove to be useful targets of existing carbohydrate vaccines and those in development.

In conclusion, we have shown that glycan microarray analysis used to identify new candidate glycan biomarkers for HCC may be useful in the screening of serum biomarkers for HCC. The biomarker candidates we identified using glycan microarray analysis showed good predictive sensitivity in the study population, and in general, they were superior to AFP. Addition of the above results to the conventional liver function tests may indeed enhance the diagnostic accuracy of liver diseases. Glycan microarray analysis provides a powerful tool for high-sensitivity and high-throughput detection of serum antibodies against CACAs, and the identified serum biomarkers may be useful for early detection of HCC in high-risk populations.

## Materials and Methods

### Ethics Statement

The study protocol was approved by the Institutional Review Board of Human Subjects Research Ethics Committee of Cheng Kung University Hospital in Taiwan (No. ER-99-176). Additionally, written informed consent was obtained from participants for the use of their blood in this study.

### Serum Samples

Serum samples from healthy individuals (n = 33), chronic HBV (n = 133), chronic HCV (n = 134) and patients with HCC (n = 293) were collected at the National Cheng Kung University Hospital in Tainan, Taiwan. Samples were encrypted to protect patient confidentiality and were used under a protocol approved by the Institutional Review Board of Human Subjects Research Ethics Committee of Cheng Kung University Hospital in Tainan, Taiwan. The patients were diagnosed using a combination of data (clinical, laboratory, and imaging findings and/or biopsy). All samples were stored at −20°C until use.

### General Methods

NEXTERION slide H were purchased from SCHOTT North America. The coating on the SCHOTT NEXTERION Slide H consists of a cross-linked, multi-component polymer layer activated with N-hydroxysuccinimide (NHS) esters to provide covalent immobilization of amine groups. The general procedure for the synthesis of glycans was conducted as reported [20], [21], [43].

### Glycan Microarray Fabrication

Microarrays were printed (BioDot; Cartesian Technologies) with a robotic pin (SMP3; TeleChem International) with a deposition of **≈** 0.7 nL of various concentrations of amine-containing glycans in printing buffer (300 mM phosphate buffer [pH 8.5] containing 0.005% Tween-20) from a 96-well microtiter plate onto NHS-coated glass slides. The slides were spotted with 50 uM solutions of each glycan, with two rows from bottom to top and five vertical replicates in each subarray. Each slide was designed for 14 grids. Printed slides were allowed to react in an atmosphere of 80% humidity for 1 h followed by desiccation overnight. The slides were stored at room temperature in a desiccator until use. Before the binding assay, the slides were blocked with ethanolamine (50 mM ethanolamine in 50 mM borate buffer [pH 9.2]) and then washed twice with water and phosphate buffer saline (PBS) (pH 7.4).

### Microarray Analysis of Serum Samples

Serum samples from patients with HCC and healthy individuals were diluted 1∶20 with a buffer consisting of 0.05% Tween 20 and 3% BSA in PBS (pH 7.4), applied to the grids of the glycan microarrays, and then incubated in a humidified chamber with shaking for 1 h. The slides then were washed three times each with 0.05% Tween 20 in PBS (pH 7.4), PBS (pH 7.4), and H_2_O. Next, Cy3-conjugated goat anti–human IgG antibody (Jackson ImmunoResearch) was added to the slides, as described above, and the slides were incubated under a coverlid in a humidified chamber with shaking for 1 h. The slides were washed three times each with 0.05% Tween 20 in PBS (pH 7.4), PBS (pH 7.4), and H_2_O, and then dried. The slides were scanned at 532 nm (for Cy3-conjugated secondary antibody) with a microarray fluorescence chip reader (arrayWoRx microarray reader).

### Data Analysis

The software GenePix Pro (Axon Instruments) was used for the fluorescence analysis of the extracted data. The local background was subtracted from the signal at each antibody spot. The “medians of ratios” from replicate spots were averaged within the same array. To obtain the relative binding intensities of glycans in human sera, we set the binding intensity for Gb5 to 100% and normalized the relative binding intensities of each glycan analog for each serum sample. The ratio of anti-glycan: anti-Gb5 in sera was calculated by dividing the mean relative binding intensity for glycan replicates by the mean relative binding intensity for Gb5 replicates, and the results were expressed as percentages. Sensitivity was calculated as the ratio of the number of HCC samples that were classified correctly as HCC (true positives) to the total number of HCC samples. Finally, statistical analysis of IgG levels in patients with HCC and healthy individuals was performed using an unpaired Student’s *t*-test. Receiver operating characteristic (ROC) curves were constructed and the area under the curve (AUC) was calculated to evaluate the specificity and sensitivity of predicting cases and controls. Data analysis was done using Prism 5 (GraphPad Software, Inc.) and SigmaPlot 9.0 software (SigmaPlot).

## Supporting Information

Figure S1 **Synthesis of glycans.** The chemistry compose method was used to synthesize 58 carbohydrate antigens.
(TIF)
