# Supplementary figures and images for: Cancer-Associated Carbohydrate Antigens as Potential Biomarkers for Hepatocellular Carcinoma
Source: PLoS One. 2012 Jul 13;7(7):e39466. doi: 10.1371/journal.pone.0039466 (PMC3396621; doi:10.1371/journal.pone.0039466)

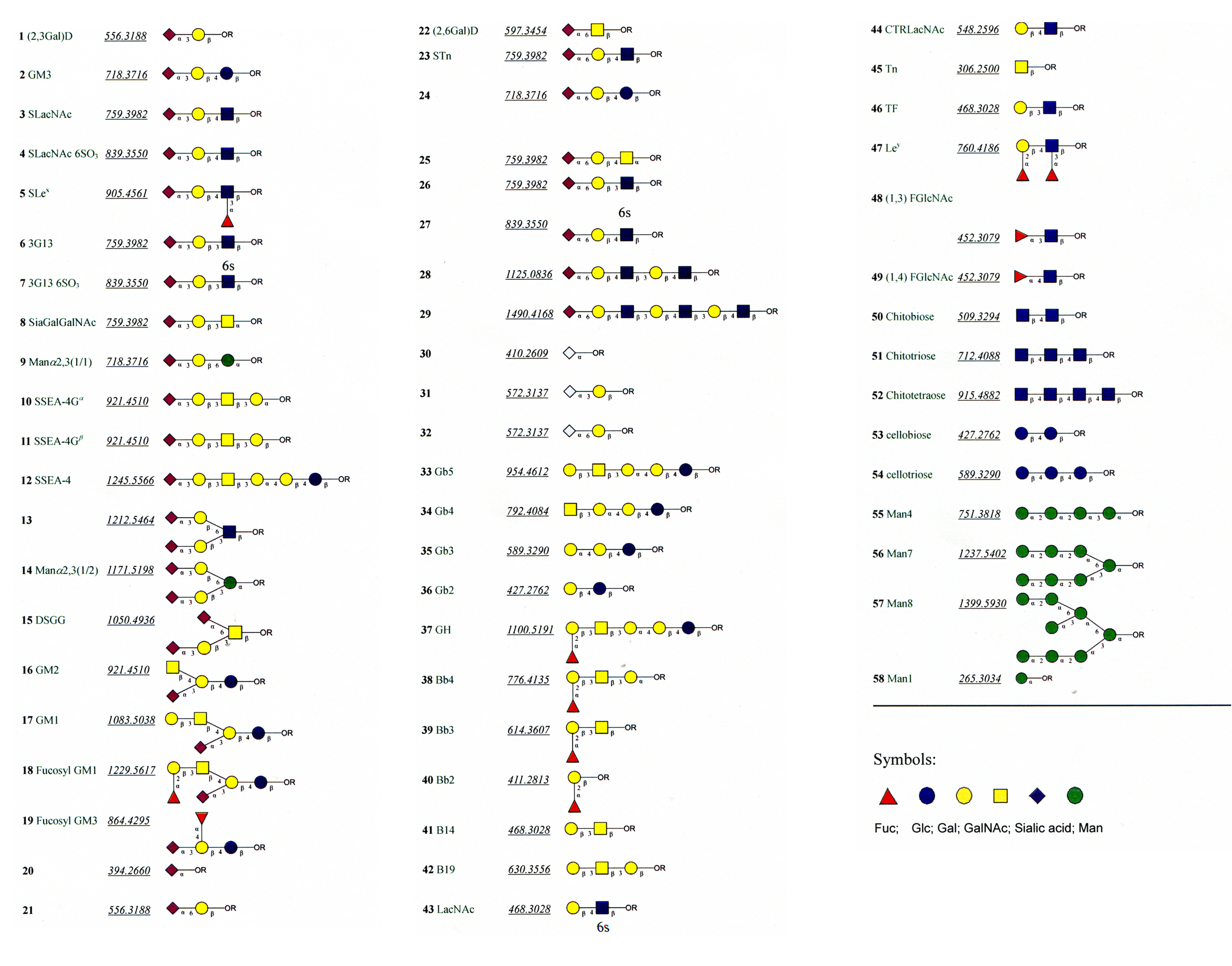

Supplement: Figure S1 — Synthesis of glycans. The chemistry compose method was used to synthesize 58 carbohydrate antigens. (TIF) [file pone.0039466.s001.tif]
